# Free vascularized tibial bone and nerve transfer: A spare-part reconstruction strategy for multiple extremity injuries^✰^

**DOI:** 10.1016/j.jpra.2026.02.006

**Published:** 2026-02-11

**Authors:** Joris Eeuwen, Vincent Stirler, Tim de Jong

**Affiliations:** a Department of Plastic, Reconstructive and Hand Surgery, Radboud University Medical Center, Geert Grooteplein Zuid 10, Nijmegen 6525 GA, the Netherlands; b Department of Surgery, Radboudumc University Medical Center, Geert Grooteplein Zuid 10, Nijmegen 6525 GA, the Netherlands; c Military Health Organisation, Ministry of Defence, Lundlaan 1, Utrecht 3584 EZ, the Netherlands

**Keywords:** Reconstructive surgical procedures, Free tissue flaps, Tibia, Peripheral nerve injuries, Upper Extremity Injuries, Amputation

## Abstract

We describe a novel reconstructive approach using a free vascularized tibial bone transfer with skin and tibial nerve components to salvage a complex upper extremity injury in the setting of a non-salvageable lower limb. This technique was applied in a patient who presented with severe blast injuries to the left upper arm and to the right upper and lower leg. Injuries of the right lower leg were so severe that reconstruction was deemed surgically not feasible. The harvested composite free flap included tibia, posterior tibial artery, tibial nerve and a skin paddle based on the posterior tibial artery. This “spare-part” salvage surgery repurposes otherwise discarded autologous tissue to restore skeletal and neural integrity at a different anatomical site. This technique broadens the reconstructive options available in orthoplastic trauma care, particularly for polytrauma patients, such as those with extremity war injuries, where multiple limbs are severely affected. We propose this flap as a potential alternative in cases where a below-knee amputation is indicated and a large, vascularized bone or composite graft is required at a separate anatomical site.

## Introduction

Large segmental bone defects of the humerus, particularly after trauma present significant reconstructive challenges. Among the available techniques, free vascularized fibular grafts (FVFG) are widely regarded as the gold standard, providing structural integrity and a reliable vascular supply that support bone healing, remodeling, and integration in compromised beds.^1^^,^^2^

In this case, the tibia was used as a graft source, rendered accessible through a below-knee amputation for a non-salvageable lower limb. This spare-part strategy enables reconstruction without additional donor-site morbidity via a vascularized osteo-neuro-cutaneous tibial flap.

## Case report and technique overview

**Figure 1:**
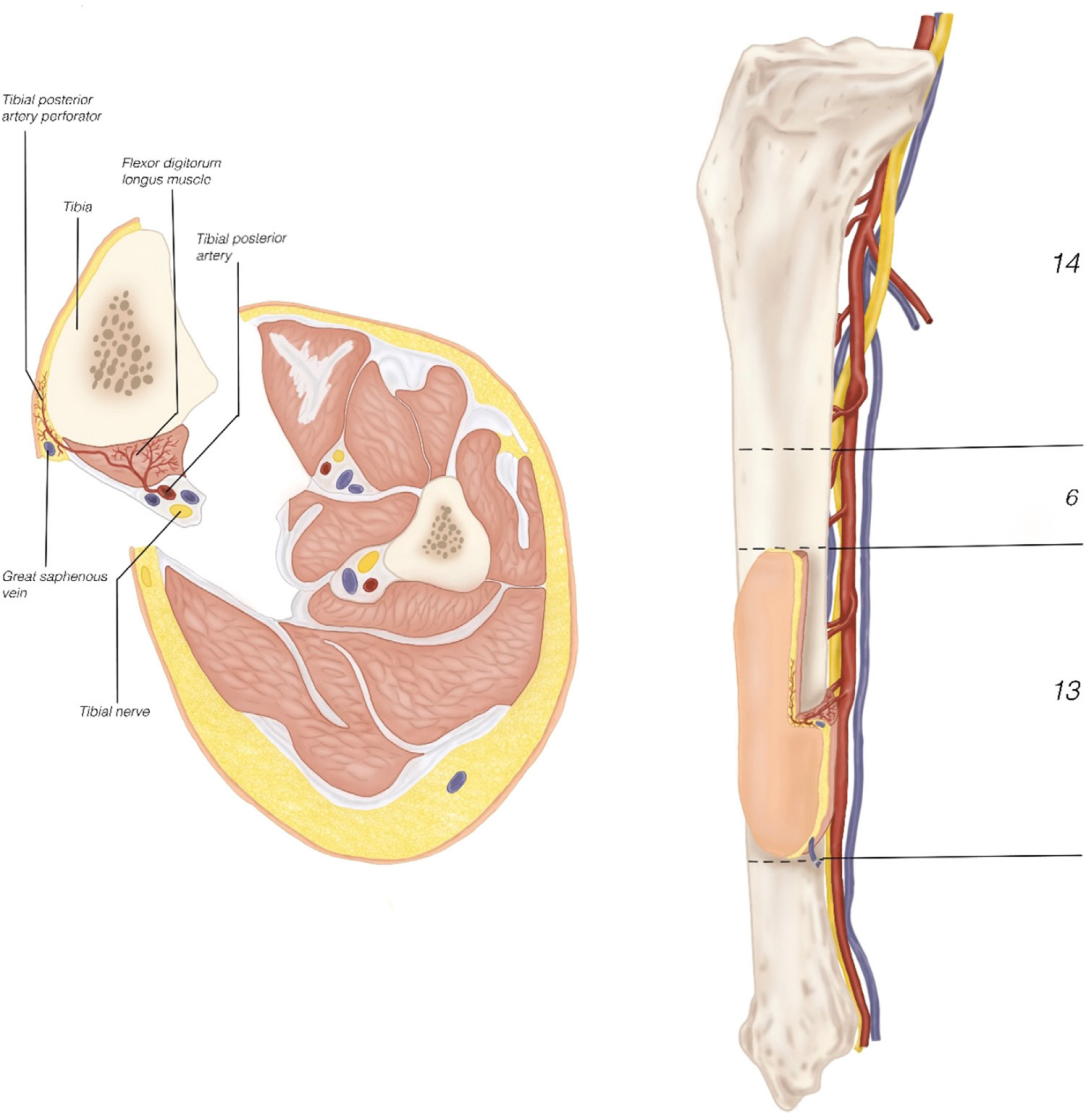
Tibial flap overview. Schematic of the vascularized osteo-neuro-cutaneous tibial flap. The tibial bone segment used for humeral reconstruction measured 13 cm, and the remaining 6 cm distal tibial segment was used as a non-vascularized graft for femur reconstruction. The posterior tibial artery supplies the tibia, flexor digitorum longus, and skin paddle; the tibial nerve runs adjacent to the pedicle, enabling en-bloc harvest.

A 52 year old male Ukrainian soldier presented with extremity war injuries of the left upper arm and the right leg following blast injury. The patient sustained a mine-blast injury on 20 October 2024, resulting in shrapnel injuries to the right femur and left shoulder. Initial management abroad consisted of serial debridement, negative pressure wound therapy, external fixation of the humerus and femur, and split-thickness skin grafting. The patient presented to our center approximately 3 months after injury. The left arm had a 13 cm diaphyseal humeral defect and a 20 cm non-functional radial nerve segment with limited lateral soft-tissue coverage. The right leg had a 10 cm femoral defect with a 10 cm sciatic nerve gap and extensive muscle damage. Additionally, the lower leg was compromised with extensive soft tissue damage including exposed fibula, pressure ulcer of the heel and complete paralysis due to sciatic nerve injury. Below-knee amputation was indicated due to factors rendering limb salvage surgically unfeasible, including a lateral lower-leg wound with fibular osteomyelitis, a heel pressure ulcer, and a 4 cm sciatic nerve defect (without neuroma excision). The treatment plan included reconstruction of the left arm (bone, nerve, skin) and right femur (bone), as well as a planned below-knee amputation on the right. A free flap was designed from the amputated lower limb segment, harvesting the tibia en-bloc with tibial nerve, and a skin padle based on the posterior tibial artery (Figure 1). Preoperative CT angiography confirmed intact posterior tibial artery anatomy without traumatic injury or anatomical variants. Graft length was planned using three-dimensional imaging of the humeral defect. Spare-part reconstruction was discussed preoperatively, and informed consent was obtained.

**Figure 2:**
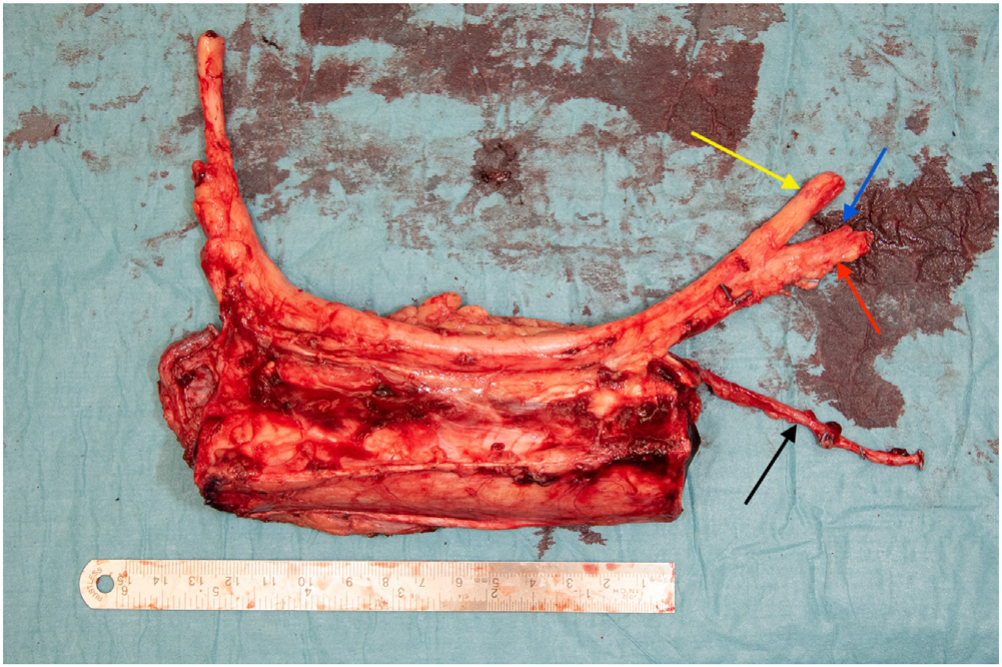
After harvest - Flap design, the tibial bone size matched to the 13 cm humeral defect. Red arrow: posterior tibial artery. Blue arrow: concomitant vein. Yellow arrow: Tibial nerve. Black arrow: great saphenous vein (VSM).

The operation commenced with removal of external fixator and exposure of the humerus via an anterolateral approach through existing scar tissue. Non-viable bone was resected resulting in a 13 cm bone defect. The radial nerve was found to be severely damaged and nonfunctional upon intraoperative testing confirming the findings of the ultrasound preoperatively. The 20 cm pathologic nerve segment was excised. Simultaneously, a vascularized tibial graft was harvested from the right lower leg, matched to the size of the humeral defect. The flap included the tibial nerve, and a skin paddle to ensure soft tissue coverage during fixation (Figure 2). A cuff of flexor digitorum longus muscle situated between the posterior tibial artery and bone was included to preserve the blood supply to the bone.

**Figure 3:**
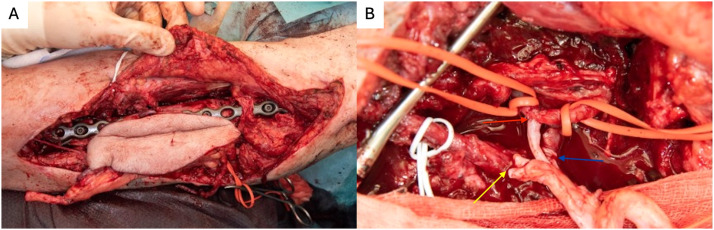
Flap inset. (A) - Humeral stabilization via plate osteosynthesis. (B) - Red arrow: microvascular end-to-side anastomosis between the posterior tibial and brachial arteries (9/0 Ethilon). Blue arrow: end-to-end venous anastomosis. Yellow arrow: radial nerve reconstruction using the tibial nerve.

The humerus was stabilized with plate osteosynthesis (Figure 3a). Microvascular anastomosis was performed between the posterior tibial artery and the brachial artery in an end-to-side configuration (Figure 3b). The brachial artery was selected as the recipient vessel due to its direct exposure within the zone of injury following complete loss of the humeral shaft. Venous outflow was established using two end-to-end anastomoses: one concomitant vein of the posterior tibial artery was anastomosed to a concomitant vein of the brachial artery, and the great saphenous vein was anastomosed to the cephalic vein using a Synovis GEM venous coupler device. The tibial nerve was used as a vascularized nerve graft to reconstruct the radial nerve. Upon completion of the upper limb reconstruction, the right lower leg was amputated below-knee leaving 14 cm of tibia and closed with a situative fish-mouth incision. A 6 cm non-vascularized tibia bone graft was used to reconstruct the distal femur bone defect. The harvested tibial segments were allocated according to defect size, with a 13 cm vascularized segment used for humeral reconstruction and the remaining 6 cm applied as a non-vascularized graft for the femur.

**Figure 4:**
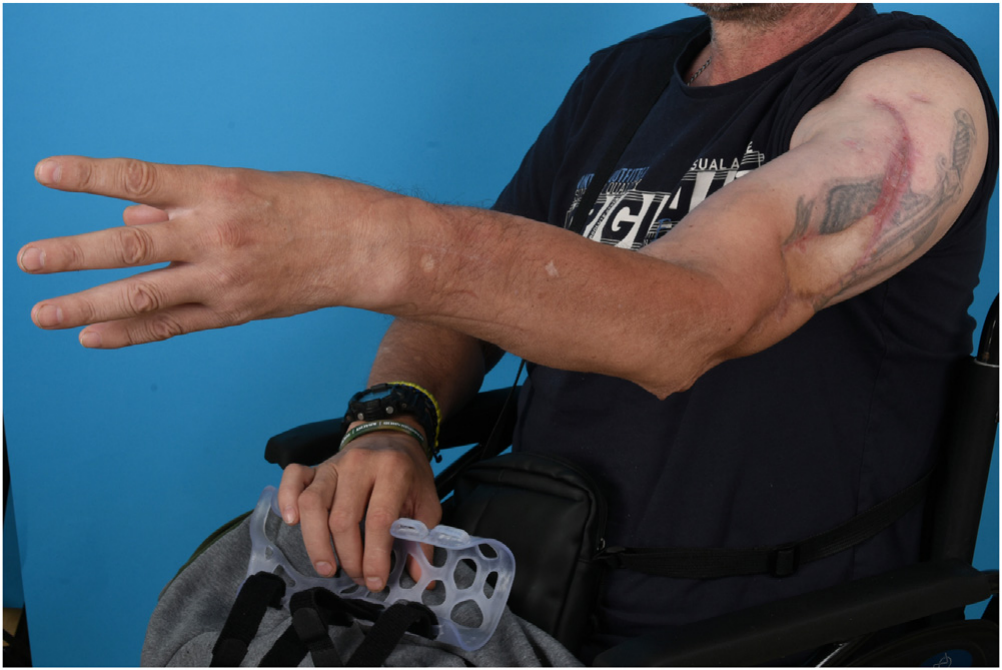
Seven-month postoperative outcome. Clinical photograph of the patient’s left arm demonstrating healed soft tissue coverage and donor tibial nerve transfer following vascularized tibia flap reconstruction.

At 7 months postoperatively, satisfactory soft-tissue healing and contour restoration of the upper arm were observed (Figure 4). Plain radiographs demonstrated maintained alignment of the graft with early signs of osseous consolidation. As neurological recovery of the reconstructed radial nerve was expected to be limited; 3 weeks later a triple tendon transfer was performed (palmaris longus to extensor pollicis longus, flexor carpi radialis to extensor digitorum communis, and pronator teres to extensor carpi radialis brevis), and sciatic nerve repair with peroneal nerve grafts.

**Figure 5:**
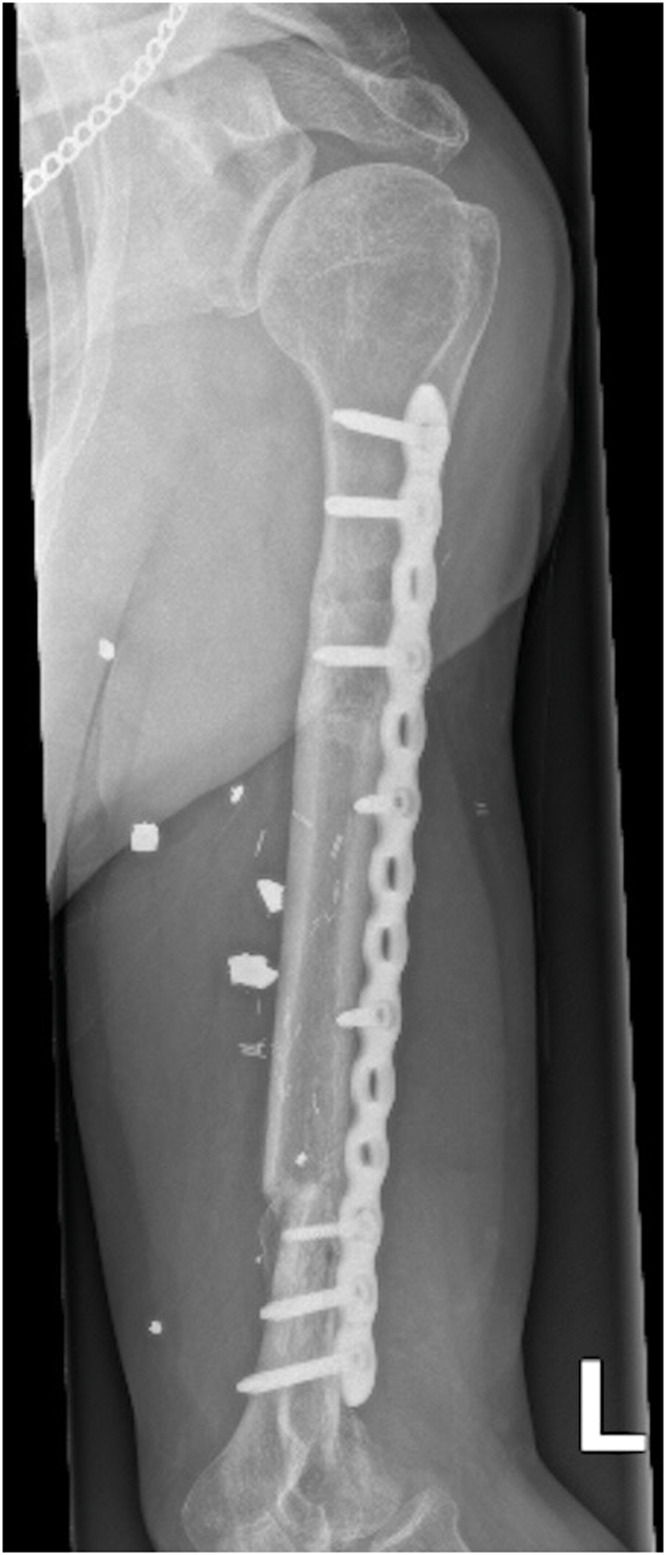
Eleven-month follow-up imaging. Single-view radiograph of the left humerus demonstrating complete consolidation of the vascularized tibial graft with maintained alignment and stable plate osteosynthesis.

At 11 months postoperatively, the patient reported no pain and was independently ambulatory using a right lower limb prosthesis. Radiographic evaluation demonstrated complete consolidation of the vascularized tibial graft with maintained alignment and stable plate osteosynthesis (Figure 5). Functional assessment showed a LEFS score of 33/80, consistent with early-stage prosthetic ambulation, and a DASH score of 35.8, indicating mild-to-moderate upper limb disability with residual thumb extension deficit and reduced performance in forceful and fine motor tasks.

## Discussion

The feasibility of this flap is supported by the anatomical understanding of tibia bone vascularization. The tibia is reliably perfused by a rich periosteal network, which provides sufficient vascular supply for cortical bone viability when carefully preserved. Nelson et al.^3^ described a dense periosteal vascular plexus with radial branches that penetrate the cortex, forming an extensive capillary bed around the bone. More recently, Levack *et al*.^4^ demonstrated through high-resolution MRI analysis that the outer third of the tibial cortex receives substantial perfusion from periosteal vessels, including the distal diaphysis. The reliability of this region for vascularized bone transfer has also been confirmed in previous reports, where tibial flaps harvested from amputated limbs were successfully used for upper and lower limb reconstruction.^5^^,^^6^ Traumatic injury to the posterior tibial artery should be considered a contraindication to this flap, underscoring the importance of preoperative vascular imaging when planning spare-part reconstruction.

The level of tibial osteotomy and preservation of a 14 cm residual tibial segment for the below-knee amputation stump was guided by contemporary evidence optimizing prosthetic fitting and stump biomechanics. A ratio-based approach, as described by Cognetti et al.,^7^ aims to balance adequate residual limb length with durable soft-tissue coverage and long-term functional outcomes.

The posterior tibial artery also plays a central role in the vascularization of the tibial nerve. Segmental branches and a central artery from this system allow for the safe inclusion of the distal two-thirds of the nerve in a neurovascular flap.^8^ This configuration makes it convenient for en-bloc harvesting. In addition to bone and nerve, the posterior tibial artery consistently perfuses the surrounding musculature of the deep posterior compartment, such as the flexor digitorum longus and tibialis posterior, as well as the overlying skin through reliable cutaneous perforators.^9^

This shared vascular territory enables the elevation of a composite flap encompassing bone, nerve, muscle, and skin, all supported by a single vascular pedicle. The anatomical and vascular coherence of this region makes the posterior tibial artery a dependable and versatile option for complex, single-pedicle composite reconstruction.^10^

Although a contralateral free vascularized fibular graft was considered, this option was avoided to prevent additional donor-site morbidity to the patient’s only remaining intact lower limb in the setting of severe polytrauma, while the tibial spare-part flap provided a better caliber match and allowed en-bloc transfer of vascularized bone, nerve and skin.

The free vascularized tibial flap leverages tissue that would otherwise be discarded during below knee or higher amputations. This approach reflects Gillies’ principle of tissue conservation, repurposing viable structures from a non-salvageable limb to restore function elsewhere, and is particularly suited for patients with war-related extremity injuries.^11^

## Conclusion

Free vascularized osteo-neuro-cutaneous tibial transfer offers a viable option for simultaneous bone and nerve reconstruction in upper extremity trauma. When harvested from a non-salvageable lower limb this approach minimizes donor site morbidity. This case demonstrates the potential of spare-part surgery in war-related polytrauma.

## Author contributions

All authors contributed to the concept, drafting, revision, and final approval of the manuscript; the schematic illustration was created by the first author.

## Ethical approval

In accordance with the guidelines of the Radboud University Medical Center, this single-patient case report using anonymized clinical information did not require formal ethics committee approval.

## Patient consent

Written informed consent for publication of clinical details and images was obtained from the patient.

## Funding

None.

## Declaration of competing interest

None declared.
